# Genetic diversity of *Echinococcus* spp. in wild carnivorous animals in Kazakhstan

**DOI:** 10.14202/vetworld.2022.1489-1496

**Published:** 2022-06-15

**Authors:** Rabiga Uakhit, Ainura Smagulova, Alfiya Syzdykova, Sarsenbay Abdrakhmanov, Vladimir Kiyan

**Affiliations:** 1 Saken Seifullin Kazakh Agrotechnical University, 62 Zhenis Avenue, Nur-Sultan 010011, Kazakhstan; 2 National Center of Biotechnology, 13/5 Qorghalzhyn Hwy, Nur-Sultan 010011, Kazakhstan

**Keywords:** Asian haplotype, corsac, echinococcosis, phylogenetics, red foxes, wolf

## Abstract

**Background and Aim:**

The study of *Echinococcus* infection among farm animals in Kazakhstan was carried out to monitor the invasion among livestock and map the data obtained. Unfortunately, there are only partial data on the study of echinococcosis among wild carnivores in Kazakhstan, which makes it difficult to conduct a comparative analysis of the epidemiological situation among wild animals. The present study aimed to estimate the genetic diversity of *Echinococcus* spp. (Leuckart, 1863) in Kazakhstan based on sequence analysis of *cytochrome c oxidase subunit 1* (*cox1*) and *dehydrogenase subunit 1* (*nad1*) of worms isolated from wild carnivorous animals wolf (*Canis lupus*), red fox (*Vulpes vulpes*) and corsac (*Vulpes corsac*).

**Materials and Methods:**

DNA from parasite tissue was used as a template for the amplification of the two mitochondrial genes *cox1* and *nad1*. Sequencing was performed according to the manual for the Seq Studio Genetic Analyzer. The multiple alignments of obtained sequences were performed using the ClustalW algorithm in Mega (v.11) software. Alignments were exported as a Nexus extension and used as input for TCS v1.21 for the identification of haplotypes. The phylogenetic analysis was constructed according to the neighbor-joining method using Mega (v.11) software.

**Results:**

Analysis of the extensiveness of echinococcosis invasion showed that 6.3% were wolves, 18.2% were corsacs, and 85% were foxes. In total, 159 adults of *Echinococcus* spp. from the three species of animals in different parts of Kazakhstan were analyzed, and 17 individual biological samples were successfully sequenced. Sequence analysis of *cox1* and *nad1* genes revealed two types of echinococcosis – *Echinococcus granulosus* in red foxes and wolves, and *Echinococcus multilocularis* in corsacs. Sequencing of a portion of the mitochondrial genome made it possible to determine seven haplotypes of the pathogen in the studied samples of *E. granulosus*. Molecular analysis of *cox1* and *nad1* genes of *E. multilocularis* revealed three new haplotypes, which have significant variability compared with other studied Asian haplotypes.

**Conclusion:**

This study made it possible to fill the gaps in understanding the localization of the foci of the spread of the echinococcosis pathogen among the main wild carnivores and to determine the species reservoir of the pathogen in the greater territory of Kazakhstan.

## Introduction

Echinococcosis is an animal-borne infection caused by tapeworms of the *Echinococcus* type [1]. Intermediate hosts for *Echinococcus* spp. are some herbivores (cattle, sheep, pigs, horses, etc.) and omnivores, such as small rodents, including rats and mice [2, 3]. The final hosts are carnivores whose intestines are inhabited by mature worms [4]. Based on the specificity of manifestation and distribution in the host’s internal organs, four types of echinococcus are distinguished: Cystic (*Echinococcus granulosus*), alveolar (*Echinococcus multilocularis*), polycystic (*Echinococcus vogeli*), and monocystic (*Echinococcus* *oligarthrus*) [5]. Cystic and alveolar echinococcoses are serious problems for medicine, public health, and animal husbandry. These two forms of echinococcosis are most common in wild animals [6, 7]. *E. granulosus* is endemic on all continents, while *E. multilocularis* has a more limited distribution [8]. In the Midwest, cystic echinococcosis (CE) is common in most countries, although the prevalence of alveolar echinococcosis has been reported in Iran, Iraq, and Tunisia [9]. In Asia, canine infections have been reported in dogs in Kazakhstan [10], Kyrgyzstan [11] and China [12]. The geographical distribution of alveolar echinococcosis is limited to the Northern Hemisphere. The cestode has been registered in the republics of Central Asia [13, 14, 15], Central Europe, the Middle East, Russia, and Alaska [14], and Northern Japan [16].

The study of *Echinococcus* infection among farm animals in Kazakhstan territory was carried out to monitor the invasion among livestock, and further map the data obtained [17, 18]. Unfortunately, there are only partial data on the study of echinococcosis among wild carnivores in Kazakhstan, which makes it difficult to conduct a comparative analysis of the epidemiological situation among wild animals.

This study aimed to study the level of echinococcal infection among wild carnivores in different regions of the country and to analyze the genetic characteristics of the echinococcosis pathogen in Kazakhstan.

## Materials and Methods

### Ethical approval

The study was approved by the Animal Ethics Committee (№1 dated July 24, 2019) of Saken Seifullin Kazakh Agrotechnical University.

### Study period and location

This study was conducted from July 2020 to February 2021. In Parasitological Laboratory, Veterinary Medicine Faculty and Research platform of Agricultural Biotechnology, Saken Seifullin Kazakh Agrotechnical University, Nur-Sultan, Kazakhstan.

### Sample collection

*Echinococcus* spp. were isolated from red foxes (*Vulpes vulpes*), corsacs (*Vulpes corsac*), and wolves (*Canis lupus*), (Linnaeus) that were captured in the different regions of Kazakhstan. Hunters conducted the extraction of wild animals in compliance with all legislative norms. The digestive tracts, livers, and lungs of 7 foxes, 11 corsacs, and 32 wolves were analyzed for the presence of parasites. The bowel scraping technique was performed as described by Deplazes and Eckert [19]. A total of 159 helminths were selected. All samples were isolated from the intestine.

### DNA extraction and polymerase chain reaction (PCR) assay

For DNA extraction, one piece of the adult worm was homogenized in an Eppendorf centrifuge tube and subjected to the standard phenol-chloroform extraction method with proteinase K and subsequent ethanol precipitation [20]. The amount and purity of the extracted DNA were determined by measuring absorption at 260 nm and 280 nm in a NanoDrop 2000 (Thermo Scientific, Carlsbad, California, USA). DNA was dissolved in ddH_2_O and stored at −70°C.

PCR was carried out in a 25 μl reaction mixture containing 10× Taq buffer with (NH_4_)_2_SO_4_, 2.5 mM MgCl_2_, 1 U Taq DNA polymerase and 200 μM deoxynucleotide triphosphates (Thermo Scientific), 10 pmol of each primer and 20 ng of extracted trematode DNA from an adult specimen as a template. Thermal cycling reactions were performed for 35 cycles of denaturation (94°C for 60 s), annealing (50°C for 60 s), and extension (72°C for 60 s). The resulting amplification fragments were separated by electrophoresis on an ethidium bromide containing 1.5% agarose gel using 1× Tris-acetate-EDTA buffer solution.

Polymerase chain reaction (PCR) was applied to identify the genetic diversity of *Echinococcus* spp. using 2 primer pairs targeting *cytochrome c oxidase subunit 1* (*cox1*: forward 5′-TTTTTTGGGCATCCTGAGGTTTAT-3′ and reverse 5′-TAAAGAAAGAACATAATGAAAATG-3′) and *dehydrogenase subunit 1* (*nad1*: forward 5′-TGGAACTCAGTTTGAGCTTTACTA-3′ and reverse 5′-ATATCAAAGTAACCTGCTATGCAG-3′) [21, 22].

### Sequencing data and phylogenetic analysis

A PCR-amplified target gene fragment was purified using a Quick PCR Purification Kit, (Invitrogen, Lithuania), following the manufacturer’s protocols. Sequencing was performed according to the manual for Seq Studio Genetic Analyzer (Thermo Fisher Scientific Applied Biosystems, USA). The resulting nucleotide sequences were visually checked by the Bio Capt program (version 11.0). The nucleotide sequences of the studied species were compared with other sequences in the National Center for Biotechnology Information (NCBI) GenBank database using the basic local alignment search tool options. The nucleotide sequences of the studied species were deposited in the NCBI GenBank database.

The genotypes of *E. granulosus* present in the isolates characterized in this study were identified based on of *cox1* gene. The original reference sequences for the G-system genotypes of *E. granulosus* described by Bowles *et al*. [20] were used as references in alignments for comparison with the sequences acquired in the present study. Multiple alignments of the obtained sequences were performed using the ClustalW algorithm in MEGA (v.11) software [23] (https://www.megasoftware.net/). Alignments were exported as a Nexus extension and used as input for TCS v1.21 (Computational Science Laboratory, Barcelona, Spain) [24] for the identification of haplotypes, network construction, and estimation of diversity indices. The phylogenetic analysis was constructed according to the neighbor-joining (NJ) method using MEGA (v.11) software. For the comparison of a relevant out-group sequence, a sequence of *E. shiquicus* was also selected.

## Results

### Parasitological study

**Figure-1:**
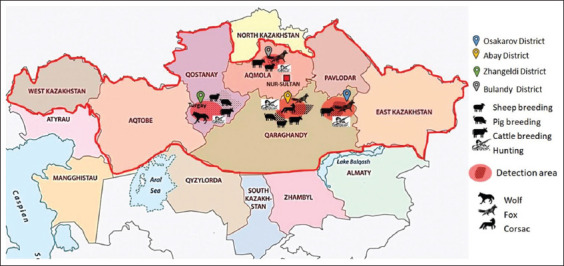
The areas in which the capture of animals was carried out and the species of animals studied from this area are depicted [Source: www.worldatlas.com/maps/kazakhstan].

All captured animals were dissected and examined for the presence of parasites. Figure-1 indicates the areas in which the capture of animals was carried out and depicts the species of animals that were studied in this area.

Echinococcosis was found in two corsacs in the Qaragandy region, and 15 specimens were isolated. Among the foxes, infected individuals were found in the Qaragandy (4 individuals) and Aqmola (2 individuals) regions. The total number of pathogens isolated from foxes was 137 specimens. Among the wolves infected with echinococcosis, two individuals from the Qostanay region were identified, and seven specimens of the pathogen were isolated from them.

The extensiveness of *Echinococcus* spp. in the Qaragandy region was 27.3% and was the highest compared to other regions in which studies have been conducted. In the Aqmola region, the extensiveness was the lowest, at 2.7%. In the present study, the exposure index values of echinococcosis were 6.3% for wolves, 18.2% for corsacs, and 85% for foxes.

### Genetic profile of E. granulosus

#### Characterization of mtDNA haplotypes

**Figure-2:**
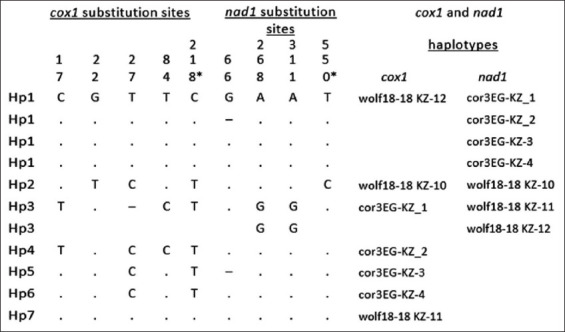
Nucleotide substitutions in the mitochondrial *cox1* and *nad1* genes, substitution sites are numbered from the initiation codon. *Synonymous substitution. Numbers are read vertically. The substitution sites are numbered from the initiation codon of each gene.

As shown in Figure-2, seven haplotypes were detected in the *E. granulosus* isolates examined, which were designated Hp1 to Hp7. When compared with individual genes, the numbers of haplotypes were decreased to 3 in *nad1* compared to 7 in *cox1*. The concatenation of the genes was effective in generating a phylogenetic tree. The nucleotide sequence of *cox1* showed the highest frequency of substitution (14 substitution sites), followed by *nad1 (*5 substitution sites).

Intraspecific variations were observed in both mitochondrial loci. Nucleotide exchanges could be observed at five and four sites in the *cox1* and *nad1* sequences, respectively, yielding 7 (*cox1*) and three (*nad1*) haplotypes (Figure-2). Most of these sites were parsimony informative. Nonsynonymous substitutions exceeded synonymous substitutions (3 vs. 1) in the *nad1* coding region, while only one of the changes in the *cox1* coding region was synonymous. Point mutation at sites 27 and 218 showed multiple evolutionary paths. Deletions were observed within both genes; hence, the nucleotide numbers were not stable.

In this study, samples of *Echinococcus* spp. from wild carnivorous animals were investigated. Data including the names of the samples, the accession numbers in GenBank, and from which animals they were isolated are presented in Table-1.

**Table 1:** *Echinococcus granulosus* genotypes and haplotypes obtained from cox1 and nad1 gene sequence analysis in different host species.

Haplotypes	Host	Genotype	Species	Accession number
Profile *cox1*				
Hp1	*Canis lupus*	G1	*Echinococcus granulosus*	OM319836
Hp2	*Canis lupus*	G1	*Echinococcus granulosus*	OM319830
Hp3	*Vulpes corsac*	G1	*Echinococcus granulosus*	MZ506656
Hp4	*Vulpes corsac*	G1	*Echinococcus granulosus*	OM469938
Hp5	*Vulpes corsac*	G1	*Echinococcus granulosus*	OM470916
Hp6	*Vulpes corsac*	G1	*Echinococcus granulosus*	OM470963
Hp7	*Canis lupus*	G1	*Echinococcus granulosus*	OM319844
Profile *nad1*				
Hp1	*Vulpes corsac*	G1	*Echinococcus granulosus*	OM640347, OM640348 OM640349, OM640350
Hp2	*Canis lupus*	G1	*Echinococcus granulosus*	OM640352
Hp3	*Canis lupus*	G1	*Echinococcus granulosus*	OM640353, OM640354

#### Parsimony network of mtDNA haplotypes

**Figure-3:**
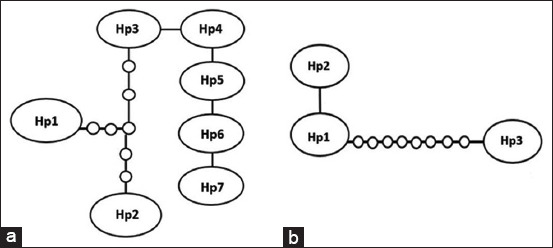
Haplotype network of the sequences of the *cox1* (a) and *nad1* (b) genes of the seven isolates from *Echinococcus granulosus*.

In general, polymorphism of the *cox1* locus resulted in seven haplotypes from the three studied regions (Figure-3a). The *nad1* locus yielded only three haplotypes, Hp1 from corsac cor3EG-KZ 1–4 in the Qaragandy region, Hp2 from one sample of a wolf in the Qostanay region (wolf18–18 KZ–10) and Hp3 from two samples from a wolf in the Qostanay region (wolf18–18 KZ–11, 12) (Figure-3b).

The haplotype diversity for *cox1* was n = 0.577778, and that for *nad1* was n = 0.644444; the number of segregating sites = 30, and Tajima’s D for *cox1* was 1.92156, while that for *nad1* was 2.06265. Large circles denote haplotypes found in this study, whereas small circles show hypothetical haplotypes.

The statistical parsimony network of the mtDNA haplotypes is illustrated in Figure-3. Among the participants in the network, *nad1* gene haplotype 3 showed the largest out-group probability from the other two haplotypes. In the *cox1* gene, haplotypes 3–7 were closely related to each other. The mutational steps within each gene clade were infrequent, and their maximum numbers were 6 in the *cox1* gene and 8 in the *nad1* gene. The network distance in the *nad1* gene between haplotypes 1 and 3 was 8 mutational steps, which indicates a small genetic divergence in a relationship. There was no dominant haplotype of *E. granulosus* in the analyzed samples.

#### Genetic differentiation index

A pairwise fixation index (Fst) was computed using the mtDNA data to estimate the degree of genetic differentiation of *E. granulosus* samples obtained from carnivorous animals in Kazakhstan. The Fst index had a 0–1 value range, where 0 denotes the complete identity of the studied samples, and 1 denotes fixation. In our analysis, the Fst values ranged from 0.002 to 0.021 (Table-2).

**Table 2:** Pairwise fixation index (Fst values) between *Echinococcus granulosus* samples based on concatenated mtDNA sequences of cox1/nad1 genes (1159 bp).

Samples	1	2	3	4	5	6	7
cor3EG-KZ_1							
cor3EG-KZ_4	0.002						
wolf18-18 KZ-10	0.005	0.002					
wolf18-18 KZ-12	0.007	0.005	0.007				
cor3EG-KZ-2	0.008	0.010	0.005	0.015			
wolf18-18 KZ-11	0.015	0.016	0.007	0.002	0.021		
cor3EG-KZ-3	0.018	0.001	0.002	0.005	0.017	0.005	

**Figure-4:**
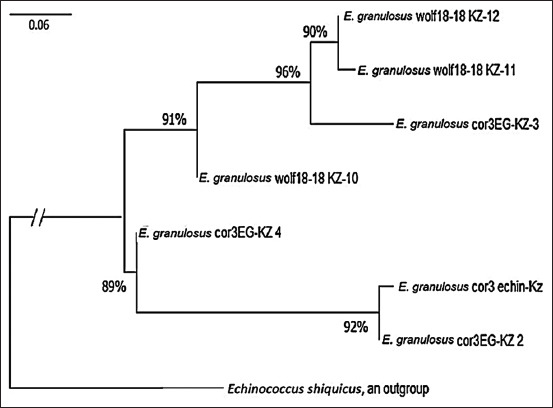
A neighbor-joining haplotype tree of *Echinococcus granulosus* constructed from the nucleotide dataset of mitochondrial *nad1* and *cox1* genes. Values on the tree nodes are bootstrap proportions (%). A scale bar (divergence of 0.06) is shown.

The evolutionary history was inferred using the NJ method [25]. The optimal tree is shown in Figure-4. The evolutionary distances were computed using the maximum composite likelihood method [23] and are expressed in units of the number of base substitutions per site. All ambiguous positions were removed for each sequence pair (pairwise deletion option). There were a total of 1159 positions in the final dataset. Evolutionary analyses were conducted in Mega 11 [26].

To analyze and build a phylogenetic tree, the genomic regions of the *cox1* and *nad1* genes were used. The percentage of relatedness between the studied genotypes was high, and the statistical assessment of the reliability of the tree obtained by bootstrap analysis ranged from 89 to 96%, forming a paraphyletic clade among themselves. According to the distance matrix, an unrooted tree was first built. For rooting, the position of the root was determined using a representative out-group; in our case, *E. shiquicus* was chosen.

### Genetic profile of *E. multilocularis*

The names of the *E. multilocularis* samples, the accession numbers in GenBank, and the types of animal parasites isolated are presented in Table-3. Phylogenic analysis was performed as described above.

**Table 3:** *Echinococcus multilocularis* genotypes and haplotypes obtained from cox1 and nad1 gene sequence analysis in different host species.

Haplotypes	Host	Species	Profile (accession number)
Hp1	*Vulpes vulpes*	*Echinococcus multilocularis*	OM640355 foxEM-KZ 1 nad1
Hp2	*Vulpes vulpes*	*Echinococcus multilocularis*	OM640356 foxEM-KZ 2 nad1
Hp3	*Vulpes vulpes*	*Echinococcus multilocularis*	OM471710 fox21-34_KZ_5 cox1

**Figure-5:**
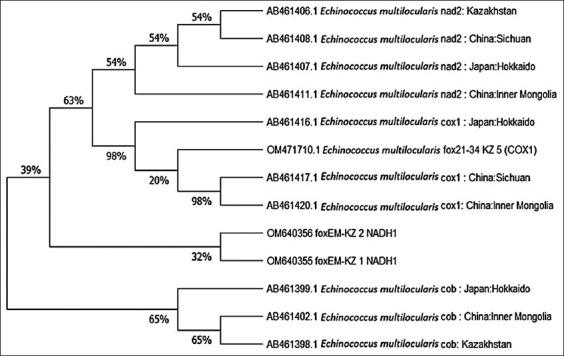
A neighbor-joining haplotype tree of *Echinococcus multilocularis*.

The resulting samples were analyzed using the Asian haplotypes of *E. multilocularis* previously described by Nakao *et al*. [27]. The phylogenetic tree was inferred using the NJ method [25]. The optimal tree is shown in Figure-5. The evolutionary distances were computed using the maximum composite likelihood method [26] and are expressed in units of the number of base substitutions per site. There were a total of 1641 positions in the final dataset.

The phylogenetic analysis (Figure-5) shows that the samples we studied had low similarity with the previously described Asian haplotypes. Given that the sample size of the studied samples was small, the study of the genetic diversity of *E. multilocularis* will continue further.

## Discussion

In this work, a study was conducted in five regions of Kazakhstan: the Qostanay, Aqmola, Qaraganda, West Kazakhstan, and East Kazakhstan regions. The territories where outbreaks of echinococcal invasion of farm animals were registered were studied. On referencing a map, it can be noted that hunting for wild animals has developed in the areas of echinococcosis detection among farm animals. This indicates the circulation of the invasion in the ecosystem and the high risks of its transmission to humans [17, 28, 29]. *Echinococcus* spp. are widespread in Kazakhstan and are responsible for massive cases of CE in humans and animals, both in dogs and livestock [30].

Comparing our data to those of neighboring states according to Beiromvand *et al*. [31], the study of carnivore parasites in Iran by multiplex PCR revealed that *E. multilocularis* was found in the feces of wild predators such as jackals, foxes, wolves, hyenas, and dogs (6.5%). *E. granulosus* was also found in fecal samples of dogs (16.9%), jackals and foxes, and wolves and hyenas (66.7%). In the Russian Federation, studies were carried out by Andrejanov [32] on the prevalence of alveolar echinococcosis among carnivores. Among the 242 animals studied, 24.5% of foxes, 50% of wolves, 18.7% of raccoon dogs, and 3.7% of domestic dogs were infested with *E. multilocularis*. Data from Rojas *et al*. [33] from Kyrgyzstan show molecular data from 43 dog samples (23 infected with *E. multilocularis* and 20 with *E. granulosus*). As a result of this study, samples of wolves and corsacs were found and established to be infected with *E. granulosus*. Foxes were infested exclusively with *E. multilocularis*. This incident is interesting and requires detailed study.

The previous studies have emphasized that the genetic variability of *E. multilocularis* is significantly limited compared to that of *E. granulosus sensu lato*, which shows significant genetic variability [21, 34, 35]. At present, ten different genotypes of *E. granulosus*, designated G1–G10, have been described worldwide based on genetic diversity associated with the nucleotide sequences of the mitochondrial *nad1* and *cox1* genes.

A total of 17 samples were sequenced and identified to the strain level in this study. Phylogenetic analysis revealed that seven isolates were identified as the G1 genotype and included seven different haplotypes (the GenBank accession numbers are shown in Table-1).

A study conducted in the territory of Kazakhstan revealed that the dominant circulating *E. granulosus* genotype was G1, which is highly pathogenic for humans and therefore for wild animals, which have the ability to easily adapt to various environmental conditions and could play a significant role as reservoirs [36].

For the first time in the territory of Kazakhstan, haplotypes of *E. granulosus* were studied. Ultimately, seven haplotypes were identified and deposited from the studied samples, which were obtained from wild carnivores. Of the two loci studied, the *cox1* site showed greater variability, dividing the studied samples into seven haplotypes. The *nad1* region, which divided the samples into only three haplotypes, was the least variable. This shows the need for further in-depth study of possible haplotypes of this species, not only those selected among wild carnivores but also domestic, farm animals, and humans.

The study of *E. multilocularis* requires more research. Using the methodology described by Nakao *et al*. [27] to analyze genetic variation within *E. multilocularis* allowed us to obtain datasets that are comparable with other sequences from different geographic regions. In our case, they were compared with Asian haplotypes. The haplotypes established by us had very low similarity with the compared samples, indicating that there is a difference and the possibility of determining a new Asian haplotype. It should also be noted that we cannot compare our dataset with different sequences, as shorter sections of mitochondrial genes were sequenced or the total lengths of different mitochondrial genes were very different.

Attempts were made to study a complete map of the distribution and transmission of invasion in the ecosystem and provide a detailed analysis and identification of all available haplotypes common in Kazakhstan. However, the work was not completed due to the lack of information available in the database. Studies of species specificity using molecular methods are practically not carried out due to the poor equipment and the technical base and the lack of adapted express protocols for the isolation and identification of parasites. These limitations negatively influence the objective assessment of the species of parasites, their identification and efficiency during analysis, etc.

The lack of an update in the direction of molecular identification of *Echinococcus* infection suggests that there is a huge gap that has implications for medicine, livestock, and hunting.

## Conclusion

These studies made it possible to fill the gaps in understanding the localization of the foci of the spread of the echinococcosis pathogen among the main wild carnivores and to determine the species reservoir of the pathogen in the greater territory of Kazakhstan. The availability of the obtained data will make it possible to understand the main ways of transmission of echinococcosis between natural reservoirs, farm animals, and humans. Together, this will contribute to the development of preventive measures to prevent the spread of echinococcosis among people and domestic animals and reduce the epidemiological background of this infection.

## Authors’ Contributions

VK: Conception and design of the study and drafted and revised the manuscript. RU: Performed DNA extraction and PCR, interpreted the data, performed phylogenetic analysis and drafted the manuscript. ASm and ASyz: Collected samples and performed parasitological isolation and typing. SA: Analyzed the data and performed the statistical analysis. All authors have read and approved the final manuscript.
